# Leveraging Immunopeptidomics To Study and Combat Infectious Disease

**DOI:** 10.1128/mSystems.00310-21

**Published:** 2021-08-03

**Authors:** Owen K. Leddy, Forest M. White, Bryan D. Bryson

**Affiliations:** a Department of Biological Engineering, Massachusetts Institute of Technology, Cambridge, Massachusetts, USA; b Koch Institute for Integrative Cancer Research, Massachusetts Institute of Technology, Cambridge, Massachusetts, USA; c Ragon Institute of MGH, MIT, and Harvard, Cambridge, Massachusetts, USA; d Center for Precision Cancer Medicine, Massachusetts Institute of Technology, Cambridge, Massachusetts, USA; NIAID, NIH

**Keywords:** host-pathogen interactions, immunology, immunopeptidomics, infectious disease, mass spectrometry, proteomics, vaccines

## Abstract

T cells must recognize pathogen-derived peptides bound to major histocompatibility complexes (MHCs) in order to initiate a cell-mediated immune response against an infection, or to support the development of high-affinity antibody responses. Identifying antigens presented on MHCs by infected cells and professional antigen-presenting cells (APCs) during infection may therefore provide a route toward developing new vaccines. Peptides bound to MHCs can be identified at whole-proteome scale using mass spectrometry—a technique referred to as “immunopeptidomics.” This technique has emerged as a powerful tool for identifying potential vaccine targets in the context of many infectious diseases. In this review, we discuss the contributions immunopeptidomic studies have made to understanding antigen presentation and T cell priming in the context of infection and the potential for immunopeptidomics to inform the development of vaccines to address pressing global health problems in infectious disease.

## INTRODUCTION

Recognition of pathogen-specific peptide-major histocompatibility complexes (pMHCs) is required for naive T cells to become activated and proliferate, and for effector T cells to recognize infected cells and carry out effector functions such as cytokine secretion and lysis of infected cells that ultimately lead to control of an infection. Antigen-specific T cell responses are also required for B cells to undergo affinity maturation and produce high-affinity antibodies (1). Identifying antigens that can be presented on MHCs and recognized by T cells is therefore essential for understanding natural immunity to infection and developing effective vaccines.

The pMHC repertoire is highly complex within any given individual, and highly variable among individuals. MHC class I and class II present peptides for recognition by CD8^+^ and CD4^+^ T cells, respectively, and are loaded with peptides via distinct pathways of antigen processing that have been reviewed in detail elsewhere (2, 3). MHC molecules are highly genetically polymorphic, and different alleles preferentially bind different sets of peptide sequences. Three distinct human leukocyte antigen (HLA) loci encode the alpha chain of MHC class I (HLA-A, HLA-B, and HLA-C), for up to six alleles in a given individual. Similarly, three loci each encode an alpha chain and a beta chain of MHC class II (HLA-DR, HLA-DQ, and HLA-DP) (4). Given the diversity and variability of the immunopeptidome, whole-proteome-scale approaches are needed to comprehensively identify pathogen-derived antigens presented on MHCs.

**FIG 1:**
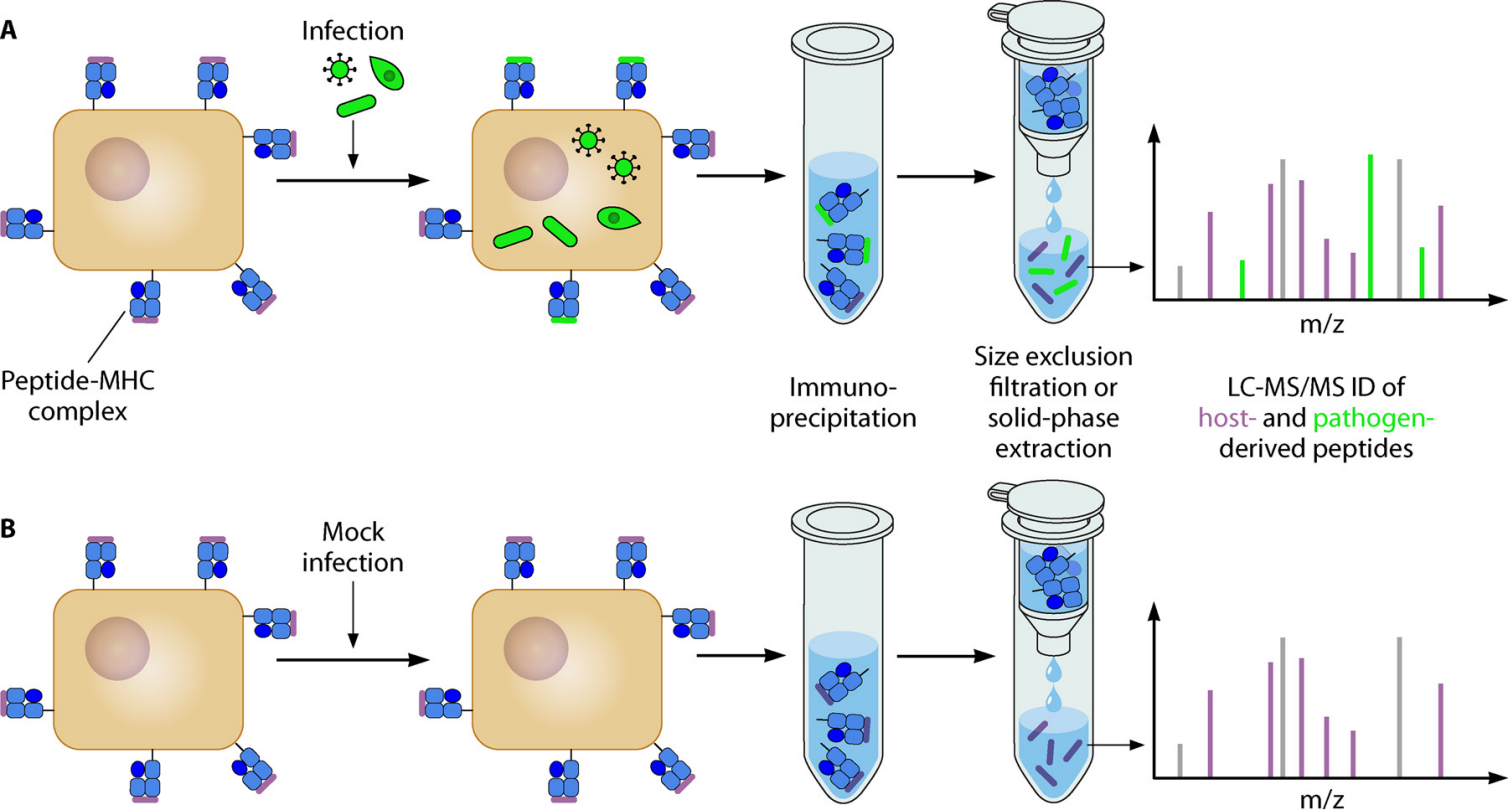
Schematic representation of an immunopeptidomic workflow to identify pathogen-derived peptides in infected cells. Infected cells and mock-infected control cells are separately lysed, lysate is subjected to immunoprecipitation with an MHC-specific antibody, and peptides are eluted in acid and separated from MHC proteins using size filtration or solid-phase extraction. Finally, the eluted peptides are analyzed by LC-MS/MS, and mass spectra are searched against a database that includes the proteomes of both the pathogen and the host to identify MHC-associated peptides. Putative pathogen-derived peptides identified in the samples from infected cells can be looked for in the data from the mock-infected control to identify and eliminate some false positives.

Mass spectrometry (MS) provides a means of experimentally identifying the repertoire of peptide antigens presented on MHCs by cells infected with a pathogen of interest. In a typical immunopeptidomic workflow, MHC-peptide complexes are immunoprecipitated (IP) from a population of cells of interest using an HLA-allele-specific antibody or pan-HLA antibody. Peptides are released by acid elution, MHCs are separated from peptides by size exclusion or solid-phase extraction, and eluted peptides are analyzed by liquid chromatography coupled to tandem MS (LC-MS/MS) (Fig. 1) (5, 6). Mass spectra are then searched against a database of possible peptides that could be derived from the host and/or pathogen proteomes to identify pathogen-derived peptides presented on MHCs.

MS analyses of MHC peptides can be carried out in two distinct modes. In data-dependent mode, peptides are detected and prioritized for tandem mass spectrum acquisition based on their abundance, without prior knowledge of the composition of the sample, enabling discovery of novel vaccine targets. In targeted mode, a predetermined list of targets is selected for acquisition, enabling studies of the mechanism and kinetics of presentation of known antigens by providing reliable detection and quantification across multiple samples.

Here, we review the use of MS-based immunopeptidomics to study antigen presentation in infectious disease. These studies reveal basic insights into the biology of antigen presentation, provide data sets for training predictive models of antigen presentation, and guide the rational selection of vaccine targets. We go on to discuss opportunities for further study in this area, technical pitfalls that remain to be overcome, and the potential for immunopeptidomics to inform vaccine development.

## ANTIGEN PRESENTATION IN MODEL SYSTEMS

Vaccinia virus infection has served as a model system for research on the basic biology of antigen presentation in viral infection. Vaccinia virus is a large, enveloped poxvirus and a relative of variola virus (the etiologic agent of smallpox). It was widely used as a vaccine against smallpox before the eradication of the disease, and some variants such as Modified Vaccinia Ankara (MVA) are being used experimentally as viral vectors for delivery of vaccine antigens (7–9). Although the immunopeptidome of vaccinia virus could provide insight into the mechanism by which it provides protection against smallpox (10), its use in basic studies of antigen presentation is due primarily to its attractiveness as a well-characterized, tractable model of viral infection.

An immunopeptidomic study of vaccinia virus-infected cells assessed whether most viral peptides presented by MHCs are immunogenic or if a large proportion are immunologically silent (11). The authors identified 170 viral peptides bound to MHC class I, over 80% of which could be recognized by T cells isolated from vaccinia virus-infected mice (11). The authors argue that their data represent evidence that most viral peptides presented on MHCs are immunogenic, but it is unclear to what extent this conclusion can be generalized beyond vaccinia virus.

Vaccinia virus infection has also been used as a model to study the kinetics of antigen presentation during viral infection and the relationship between pMHC abundance and immunogenicity. Croft et al. (12) used a targeted MS approach to monitor presentation of eight known vaccinia virus peptides in infected mouse B cells over time. They found that peptide presentation occurred almost immediately after initiation of viral gene expression, consistent with cotranslational antigen processing (13, 14). They also found that comparably immunogenic peptides can vary widely in their abundance in the MHC repertoire, suggesting that pMHC abundance may not strongly correlate with immunogenicity. Using targeted MS to monitor antigen presentation over time could be useful in selecting vaccine antigens presented during different stages of infection (15).

## COMPUTATIONAL MODEL BUILDING

Computational methods for predicting peptides likely to be presented on MHCs were first trained using data from *in vitro* binding assays (16–19), but recently developed models incorporate large MS-based immunopeptidomic data sets as training data, resulting in improvements in performance (20–24). The high-throughput nature of *in silico* predictions enables such analyses to cover a much wider range of HLA alleles. Computational methods can also select sets of epitopes that maximize coverage of a population with a given set of HLA allele frequencies (25), which will aid in the design of broadly protective vaccines.

Immunopeptidomic data sets have facilitated the development of models that explicitly take into account factors beyond MHC binding affinity alone, such as gene expression level, protein subcellular localization, and predicted protease cleavage sites (22–24, 26). These modeling approaches could be applied to learn pathogen-specific rules for antigen presentation, such as whether secreted bacterial proteins are preferentially presented on MHCs, relative to proteins in the pathogen’s cytosol (27). Existing data sets of pathogen-derived MHC peptides are small, but highly data-efficient machine learning methods (28) may help build pathogen-specific models from limited numbers of training examples.

Rapid computational predictions can inform efforts to develop vaccines for emerging infectious diseases sooner than experimental data. Multiple computational analyses of predicted T cell epitopes in the SARS-CoV-2 proteome were published early in the pandemic (29, 30), whereas experimental data identifying MHC peptides derived from SARS-CoV-2 were not published until months later (31, 32). Obtaining accurate predictions of T cell epitopes could therefore be critical to rapidly developing vaccines against future emerging pathogens.

## VACCINE TARGET DISCOVERY

The direct discovery of vaccine targets is one of the most exciting applications of immunopeptidomics in infectious disease. Whereas T cell responses contribute to protective immunity against many globally important infectious diseases, most rationally designed vaccines licensed to date have been designed to elicit protective immunity primarily through humoral immune responses (33). Immunopeptidomics can provide a systematic, rational basis on which to select targets for vaccines that elicit protective cell-mediated immunity, which may be essential in order to develop highly effective vaccines against pathogens for which none yet exist.

Developing effective vaccines against intracellular bacteria has historically proven challenging, in part due to a lack of knowledge of protective T cell antigens (34). Immunopeptidomic analyses have helped to address this problem by identifying protective T cell epitopes of Mycobacterium tuberculosis—the causative agent of tuberculosis (35)—and Chlamydia trachomatis—the causative agent of chlamydia (36) and trachoma (37). M. tuberculosis-derived peptides have been identified that are presented on MHC class I (38) and on the noncanonical class Ib MHC molecule HLA-E (39) by M. tuberculosis-infected human cells, as well as MHC class I and II peptides presented by human macrophages infected with the bacillus Calmette-Guérin (BCG) vaccine strain (40). Administering these antigens to mice using the ChAdOx1 viral vector in a prime-boost regimen with BCG reduced M. tuberculosis bacterial burden in the lung significantly more than did BCG alone (40). Similarly, nine antigens identified in an immunopetidomic analysis of C. trachomatis-infected cells (41) were recognized by T cells of infected mice and reduced vaginal shedding of C. trachomatis when delivered as a recombinant protein vaccine (42). These findings suggest that immunopeptidomics can help advance the development of effective vaccines against intracellular bacterial pathogens, though the clinical utility of these vaccine targets has yet to be tested in humans.

Like intracellular bacteria, eukaryotic parasites have proven difficult to vaccinate against, with only one vaccine currently licensed (43). Mou et al. (44) used MS to identify peptides presented on MHC class II by murine macrophages infected with Leishmania major, a parasite that causes leishmaniasis (45). They identified an immunodominant epitope of phosphoenolpyruvate carboxykinase (PEPCK) that reduced the parasite burden in a mouse model of leishmaniasis when administered as a peptide vaccine or DNA vaccine. These results demonstrate the potential for immunopeptidomics to aid in the development of vaccines against intracellular parasites.

Immunopeptidomics may help identify viral T cell epitopes that vaccines can target to confer lasting protection in the face of viral evasion of antibody-mediated immunity. A high frequency of escape mutations (46) has made it difficult to develop vaccines that elicit antibody-mediated protection against human immunodeficiency virus (HIV)—the causative agent of AIDS (47). However, some individuals naturally control HIV infection in a manner associated with CD8^+^ T cell responses, suggesting T cells can contribute to durable immunity against HIV (48–51). Several research groups have used immunopeptidomic methods to identify HIV-derived epitopes presented on MHC class I by infected CD4^+^ T cells (52–56). Some of these studies specifically isolated peptides bound to HLA alleles associated with improved control of HIV infection (53, 55), thereby identifying antigenic targets that may be associated with durable protection. Many of these epitopes elicited gamma interferon (IFN-γ) production in T cells of HIV-positive patients, validating their relevance to T cell responses against HIV. Including additional viral T cell epitopes in future HIV vaccine candidates could help mitigate the effects of viral escape mutations, but this concept has not been tested in animal models or humans.

## INFECTION AND AUTOIMMUNITY

Some infections are known to be associated with the onset of autoimmune disease, but the mechanisms underlying this relationship are not fully understood (57). Immunopeptidomics offers a way to systematically identify pathogen-derived MHC peptides that can trigger self-reactive T cell responses, leading to autoimmunity. For example, Alvarez-Navarro et al. (58) used immunopeptidomics to investigate why Chlamydia trachomatis infection is associated with reactive arthritis in individuals carrying HLA-B alleles of the HLA-B*27 group (59). They identified three C. trachomatis peptides presented by HLA-B*27 that had high sequence similarity to endogenous human peptides and were predicted to adopt similar conformations when bound to HLA-B*27. T cells that respond to these C. trachomatis peptides might cross-react with endogenous peptides, leading to autoimmunity. Similarly, Wang et al. (60) used immunopeptidomics to reveal a mechanism that may explain why the HLA-DR*15 haplotype and Epstein-Barr virus (EBV) infection are both associated with increased risk of multiple sclerosis. They identified peptides presented by HLA-DR*15 derived from the neuronal marker RASGRP2 and found that T cells specific for these peptides cross-reacted with peptides derived from EBV. These results suggest that EBV infection may lead to T cell responses that cross-react with neuronal markers in individuals expressing HLA-DR*15, leading to neurodegeneration. Cross-reactive epitopes identified in immunopeptidomic studies could in principle be targeted by therapies designed to induce epitope-specific immune tolerance (61, 62) and mitigate autoimmunity associated with infection.

## FUTURE DIRECTIONS AND OUTSTANDING CHALLENGES

Immunopeptidomics can elucidate the basic biology and mechanisms of antigen presentation in infectious disease, improve predictive modeling of pathogen-derived T cell epitopes, draw connections between infection and autoimmunity, and identify promising vaccine targets. The discovery of new T cell antigens would aid in the development of vaccines against several globally important pathogens. For example, liver-resident CD8^+^ T cells are known to be a strong correlate of immunity to malaria (63, 64), but only a few T cell antigens presented in liver-stage malaria have been identified (65, 66). T cell responses are also thought to be important for immunity to bacterial pathogens with a rapidly growing incidence of antibiotic resistance, such as Salmonella spp. (67) and Staphylococcus aureus (68), as well as parasites such as Trypanosoma cruzi, which causes Chagas disease (69). Some of these protective T cell responses could target ligands that standard immunopeptidomic workflows do not detect, such as noncanonical translation products (70) or small molecules presented by the HLA-like molecules CD1 (71) and MR1 (72), but methods are being developed to analyze these unconventional epitopes by MS as well (70, 73, 74). The use of immunopeptidomics to comprehensively identify T cell antigens could therefore help combat both long-standing global health problems and emerging crises.

Although immunopeptidomics can provide valuable information about the repertoire of pathogen-derived MHC ligands at a whole-proteome scale, it currently has at least three important limitations, enumerated in Table 1. Some of these limitations can be overcome by combining MS-based immunopeptidomics with other complementary techniques, and some may be overcome through ongoing method development.

**TABLE 1:** Three significant limitations of current immunopeptidomic methods

	Limitation of immunopeptidomics	Implications	Relevant references
1	Not all peptides presented on MHCs are immunogenic	Targets identified by immunopeptidomics should ideally be further screened for T cell recognition and immunogenicity using other assays.	75 – 79
2	Cell-to-cell heterogeneity in antigen presentation cannot be resolved	Some contributions to the MS signal in an immunopeptidomic study may come from uninfected bystander cells, and a mixture of different cell types cannot be deconvoluted to learn which types are important for antigen presentation. Presentation of specific antigens can be probed at a single-cell level using pMHC-specific antibodies or biotin labels transferred between cells.	80 – 85
3	Large amounts of sample input are required	Studies in primary cells or with pathogens that cannot be cultured in large quantities are currently difficult to carry out. Microfluidic methods and other low-volume sample-handling techniques may help overcome these limitations. Targeted MS analyses may have higher sensitivity and require lower input.	6, 86

Determining whether a peptide discovered through immunopeptidomics is immunogenic (problem 1) generally requires combining immunopeptidomics with techniques that can directly measure a T cell response against a given peptide of interest. Traditionally, this has been done by measuring production of IFN-γ by T cells specific for each epitope of interest (40). T cells specific for a pMHC complex of interest can also be stained using pMHC tetramers (75). More recently, a suite of methods for high-throughput measurement of T cell receptor (TCR)-pMHC interactions has been developed (76). Computational models have also been developed to predict the immunogenicity of putative T cell epitopes (77–79), which may help prioritize hits identified in an immunopeptidomic experiment for development as potential vaccine targets.

Cell-to-cell heterogeneity in antigen presentation (problem 2) may be consequential for immunity to some infections. For example, certain antigens of Mycobacterium tuberculosis can be presented by both infected cells and uninfected bystander cells (80, 81), while other antigens may be specific to infected cells. An antigen presented by uninfected bystander cells could potentially cause off-target lysis of bystander cells by cytotoxic T cells if targeted by a vaccine and/or might be presented at a lower level on infected cells than immunopeptidomic data would suggest, leading to less effective targeting of infected cells. Antibodies that recognize specific peptide-MHC complexes (82, 83) can enable measurements of antigen presentation at the single-cell level using flow cytometry or microscopy. Interactions between T cells and antigen-presenting cells (APCs) can also be tracked using a transferable biotin label (84, 85), providing a single-cell readout of pMHC-TCR interactions. These methods can complement immunopeptidomics by probing antigen presentation in a heterogeneous population of cells.

Whereas recent immunopeptidomic studies in cancer immunology have used sample input on the order of 10^6^ to 10^7^ cells (6), sample input on the order of 10^8^ to 10^9^ cells (problem 3) is often required to detect pathogen-derived peptides. The use of microfluidic devices or other low-volume sample handling techniques (86) could help reduce input requirements dramatically while retaining high sensitivity. Serial immunoprecipitation has already been successfully used to isolate both MHC class I and class II peptides from the same sample, further conserving input material (40). Lower sample input requirements could enable immunopeptidomic studies on infected primary cells or *ex vivo* samples from animals infected with a pathogen of interest or studies of pathogens that are impossible to culture in large quantities, such as the liver stage of malaria parasites (*Plasmodium* spp.).

Combining computational modeling with targeted mass spectrometry approaches could provide another avenue toward reducing sample input requirements (problem 3). Targeted MS analyses can detect MHC-associated peptides with greater sensitivity than data-dependent analyses, potentially reducing sample input requirements and increasing the likelihood of detecting peptides that would not be detectable in a data-dependent analysis. Accurate predictive models of peptide presentation on MHCs could identify promising candidates to experimentally validate using highly sensitive targeted MS workflows, and these experimental results could in turn be used for further model refinement.

Future improvements in immunopeptidomic methods may also focus on overcoming technical pitfalls that can result in inaccurate identification of MHC peptides by MS. In one instance, for example, HIV-derived peptides previously identified as MHC class I ligands (52) were shown to nonspecifically copurify with host membrane proteins (87). This result highlights the importance of proper controls in immunopeptidomic studies. Ambiguities in peptide identification may also be a source of uncertainty. It has recently been proposed that posttranslationally spliced peptides (88–91) and peptides derived from noncanonical translation products (92, 93) contribute substantially to the immunopeptidome. However, matching mass spectra against an expanded search space that includes spliced peptides and noncanonical translation products may increase the risk of false positives (70, 94). The false-discovery rate associated with these identifications must be carefully estimated to obtain accurate results (70).

Immunopeptidomics has already been used to design vaccines that improve control of infection in mouse models of chlamydia (42), leishmaniasis (44), and tuberculosis (40). As the field continues to advance, it is likely that immunopeptidomics and/or computational models trained on immunopeptidomic data will provide a rapid, sensitive, and systematic means of identifying vaccine targets in many human pathogens. Combining immunopeptidomics with other next-generation immunoassays and existing preclinical and clinical vaccine development platforms has the potential to have a transformative impact on global health.
